# Human *NCR3* gene variants rs2736191 and rs11575837 alter longitudinal risk for development of pediatric malaria episodes and severe malarial anemia

**DOI:** 10.1186/s12864-023-09565-1

**Published:** 2023-09-13

**Authors:** Clinton O. Onyango, Qiuying Cheng, Elly O. Munde, Evans Raballah, Samuel B. Anyona, Benjamin H. McMahon, Christophe G. Lambert, Patrick O. Onyango, Kristan A. Schneider, Douglas J. Perkins, Collins Ouma

**Affiliations:** 1 Department of Biomedical Sciences and Technology, School of Public Health and Community Development, Maseno University, Maseno, Kenya; 2 University of New Mexico-Kenya Global Health Programs, Kisumu and Siaya, Kenya; 3 Center for Global Health, Internal Medicine, University of New Mexico, Albuquerque, NM USA; 4 Department of Clinical Medicine, School of Health Science, Kirinyaga University, Kerugoya, Kenya; 5 Department of Medical Laboratory Sciences, School of Public Health Biomedical Sciences and Technology, Masinde Muliro University of Science and Technology, Kakamega, Kenya; 6 Department of Medical Biochemistry, School of Medicine, Maseno University, Maseno, Kenya; 7 Theoretical Biology and Biophysics Group, Theoretical Division, Los Alamos National Laboratory, Los Alamos, NM USA; 8 Department of Zoology, School of Physical and Biological Sciences, Maseno University, Maseno, Kenya; 9 Department Applied Computer- and Bio-Sciences, University of Applied Sciences Mittweida, Mittweida, Germany

**Keywords:** *Plasmodium falciparum*, Malaria, Severe malaria anemia (SMA), Natural cytotoxicity-triggering receptor 3 gene (*NCR3*), Genotypes, And haplotypes

## Abstract

**Background:**

*Plasmodium falciparum* malaria is a leading cause of pediatric morbidity and mortality in holoendemic transmission areas. Severe malarial anemia [SMA, hemoglobin (Hb) < 5.0 g/dL in children] is the most common clinical manifestation of severe malaria in such regions. Although innate immune response genes are known to influence the development of SMA, the role of natural killer (NK) cells in malaria pathogenesis remains largely undefined. As such, we examined the impact of genetic variation in the gene encoding a primary NK cell receptor, natural cytotoxicity-triggering receptor 3 (NCR3), on the occurrence of malaria and SMA episodes over time.

**Methods:**

Susceptibility to malaria, SMA, and all-cause mortality was determined in carriers of *NCR3* genetic variants (i.e., rs2736191:C > G and rs11575837:C > T) and their haplotypes. The prospective observational study was conducted over a 36 mos. follow-up period in a cohort of children (*n* = 1,515, aged 1.9–40 mos.) residing in a holoendemic *P. falciparum* transmission region, Siaya, Kenya.

**Results:**

Poisson regression modeling, controlling for anemia-promoting covariates, revealed a significantly increased risk of malaria in carriers of the homozygous mutant allele genotype (TT) for rs11575837 after multiple test correction [Incidence rate ratio (IRR) = 1.540, 95% CI = 1.114–2.129, *P* = 0.009]. Increased risk of SMA was observed for rs2736191 in children who inherited the CG genotype (IRR = 1.269, 95% CI = 1.009–1.597, *P* = 0.041) and in the additive model (presence of 1 or 2 copies) (IRR = 1.198, 95% CI = 1.030–1.393, *P* = 0.019), but was not significant after multiple test correction. Modeling of the haplotypes revealed that the CC haplotype had a significant additive effect for protection against SMA (i.e., reduced risk for development of SMA) after multiple test correction (IRR = 0.823, 95% CI = 0.711–0.952, *P* = 0.009). Although increased susceptibility to SMA was present in carriers of the GC haplotype (IRR = 1.276, 95% CI = 1.030–1.581, *P* = 0.026) with an additive effect (IRR = 1.182, 95% CI = 1.018–1.372, *P* = 0.029), the results did not remain significant after multiple test correction. None of the *NCR3* genotypes or haplotypes were associated with all-cause mortality.

**Conclusions:**

Variation in *NCR3* alters susceptibility to malaria and SMA during the acquisition of naturally-acquired malarial immunity. These results highlight the importance of NK cells in the innate immune response to malaria.

**Supplementary Information:**

The online version contains supplementary material available at 10.1186/s12864-023-09565-1.

## Background

*Plasmodium falciparum,* the most prevalent malaria parasite in sub-Saharan Africa, causes 99% of the estimated malaria cases in the region [1]. The burden of malaria largely impacts children under 5 years of age, accounting for 80% of all malaria-related mortality [1]. Life-threatening complications of malaria include hyper-parasitemia, hypoglycemia, hyper-lactatemia, kidney failure, metabolic acidosis, cerebral malaria, severe malarial anemia [SMA, hemoglobin (Hb) < 5.0 g/dL with peripheral parasitemia], and respiratory distress [2]. In holoendemic *P. falciparum* transmission regions, such as western Kenya, severe malaria presents primarily as SMA [3, 4]. Despite malaria control efforts in the region, SMA has remained one of the major causes of morbidity and mortality in children aged less than 5 years [4, 5].

The selective pressure of malaria on the human genome is epitomized by the sickle hemoglobin (HbS) allele of hemoglobin beta gene (*HBB*) for which heterozygous carriers are protected against severe malaria in areas of high malaria prevalence [6, 7]. Our previous studies in western Kenya illustrate that genetic variants, particularly those in innate immune response genes, influence susceptibility to malaria and subsequent development of SMA [8–15]. Previous linkage analysis in different ethnic groups from Africa identified a linkage between the 6p21.3 locus of the major histocompatibility complex (MHC) region and susceptibility to mild malaria [16–18]. Centrally located in the MHC region is the natural cytotoxicity-triggering receptor 3 (*NCR3*). A variant located in the promoter region of *NCR3*, rs2736191:C > G, has been associated with increased mild malaria episodes in carriers of the mutant allele (C allele, which corresponds to G allele in the current study) in Burkina Faso and Congo [19, 20]. The wildtype allele of rs2736191 was found to enhance *NCR3* promoter activity [20]. Investigation of the rs2736191 polymorphism in a Senegalese population revealed no relationship between the SNP and cerebral malaria [21].

Although the role of NCR3 in the pathogenesis of SMA remains elusive, it is known that this protein is constitutively expressed on the surface of natural killer (NK) cells and plays an important role in NK cell activation, degranulation, and cytotoxicity [22, 23]. Moreover, NCR3 recognizes parasitized red blood cells (pRBCs), and directly binds to the Duffy Binding Like (DBL1-α) domain of *P. falciparum* erythrocyte membrane protein-1 (*Pf*EMP-1) without the requirement of accessory molecules, such as MHC class I [24]. Binding of pRBCs to NCR3 on NK cells also activates cell cytotoxicity and promotes the release of cytokines and chemokines known to influence the pathogenesis of SMA [25–29].

Both rs2736191 and another *NCR3* variant rs11575837:C>T (located in the non-coding region of exon 1) have been shown to be inversely associated with risk of primary Sjögren’s syndrome (pSS), and the minor T allele of rs11575837 is associated with reduced NCR3 gene transcription [30, 31]. Similar to severe malarial anemia, the pathogenesis of pSS involves immune dysregulation.

To extend previous studies on the potential role of NK cells in the pathogenesis of malaria, we examined the relationship between *NCR3* variants (rs2736191 and rs11575837) and longitudinal clinical outcomes (i.e., malaria and SMA episodes) in a cohort of Kenyan children residing in a holoendemic *P. falciparum* area. Here, we report the impact of the two *NCR3* variants on susceptibility to pediatric malaria and SMA throughout a 36-mos. follow-up period during the development of naturally-acquired malarial immunity.

## Results

### Clinical, demographic, and laboratory characteristics of the study participants at enrollment

The enrollment characteristics of the cohort, stratified according to presence of malaria and malarial anemia status [aparasitemic (*n* = 289), non-SMA (*n* = 962) and SMA (*n* = 264)], are presented in Table 1. The sex ratio was comparable among the three groups (*P* = 0.752), while age differed across the groups (*P* = 0.003) with SMA patients being the youngest. Children with SMA exhibited the lowest hematocrit levels as well as RBC counts (*P* < 0.001 and *P* < 0.001, respectively). White blood cell counts progressively increased across the groups (*P* < 0.001) and were highest in children with SMA. The non-SMA group presented with a higher parasite density than the SMA group (*P* = 0.043).

**Table 1:** Clinical, demographic, and laboratory characteristics of study participants

**Characteristics**	**Total**	**Aparasitemic (MPS Negative)**	**non-SMA (Hb ≥ 5.0 g/dL)**	**SMA (Hb < 5.0 g/dL)**	***P-*value**
**No. of participants**	**1,515**	**289**	**962**	**264**	
Sex [n, (%)]: Male	760	145 (50.2)	488 (50.7)	137 (51.9)	0.752^a^
Female	755	144 (49.8)	474 (49.3)	127 (48.1)	
Age, months	1,515	11.0 (13.4)	12.7 (10.4)	10.1 (10.7)	**0.003**^**b**^
Axillary temperature, °C	1,503	37.0 (1.5)	38.0 (1.5)	38.0 (1.1)	**< 0.001**^**b**^
**Hematological parameters**					
Hemoglobin, g/dL	1,508	10.3 (2.8)	7.7 (2.8)	4.3 (1.1)	N/A
Hematocrit, %	1,507	32.9 (8.2)	25.2 (9.1)	14.1 (3.8)	**< 0.001**^**b**^
Red Blood Cells, × 10^6^/µL	1,504	4.7 (1.1)	3.8 (1.4)	1.9 (0.6)	**< 0.001**^**b**^
White Blood Cells, × 10^3^/µL	1,503	11.0 (7.3)	11.7 (6.5)	14.9 (9.8)	**< 0.001**^**b**^
**Parasitological Indices**					
Parasite density, MPS/µL	1,226	0.0 (0.0)	28,595 (79,256)	24,831 (66,014)	**0.043**^**c**^
**Co-infections**					
HIV-1: Negative [n, (%)]	1,457	275 (95.8)	933 (97.2)	249 (94.7)	0.116^a^
Positive [n, (%)]	53	12 (4.2)	27 (2.8)	14 (5.3)	
Bacteremia: Negative [n, (%)]	1,402	257 (89.9)	902 (94.2)	243 (92.0)	**0.036**^**a**^
Positive [n, (%)]	106	29 (10.1)	56 (5.8)	21 (8.0)	
**Genetics Variants**					
α^3.7^-thalassemia: αα/αα [n, (%)]	554	105 (42.9)	355 (42.4)	94 (39.7)	**0.014**^**a**^
-α/αα [n, (%)]	505	75 (30.6)	329 (39.3)	101 (42.6)	
-α/-α [n, (%)]	261	65 (26.5)	154 (18.4)	42 (17.7)	
G6PD: Normal [n, (%)]	1,078	194 (75.8)	678 (75.8)	206 (80.5)	0.387^a^
Intermediate [n, (%)]	266	51 (19.9)	178 (19.9)	37 (14.5)	
Deficiency [n, (%)]	63	11 (4.3)	39 (4.4)	13 (5.1)	
Sickle cell trait: Hb AA [n, (%)]	1,241	223 (79.1)	785 (82.5)	233 (90.7)	**< 0.001**^**a**^
Hb AS [n, (%)]	239	55 (19.5)	164 (17.2)	20 (7.8)	
Hb SS [n, (%)]	10	4 (1.4)	2 (0.2)	4 (1.6)	

Data are presented as median (interquartile range, IQR) and [n (%)] unless stated otherwise. Children (*n* = 1,515) were categorized into aparasitemic controls (*n* = 289; no parasitemia), and either non-SMA (*n* = 962; Hb ≥ 5.0 g/dL) or SMA (*n* = 264; Hb < 5.0 g/dL). Non-SMA and SMA patients are collectively categorized as parasitemic (*n* = 1,226)

*P*-values ≤ 0.05 were considered significant and are indicated in bold

*Abbreviations*: *MPS* Malaria parasites, *HIV* Human immunodeficiency virus, *G6PD* Glucose-6-phosphate dehydrogenase deficiency

^a^Statistical significance was determined by Chi-square analysis

^b^Statistical significance determined by the Kruskal-Wallis test

^c^Statistical significance determined by Mann-Whitney U test

HIV-1 status and bacteremia were assessed in the cohort since we have shown previously that they enhance the risk of SMA [32, 33]. The distribution of HIV-1 was comparable across the groups (*P* = 0.116), whereas bacteremia significantly differed (*P* = 0.036) and was highest in the aparasitemic group. Examination of genetic factors known to influence the development of severe malaria [34–36] revealed that the distribution of α^3.7^-thalassemia variants and sickle cell trait (HbAS) differed across the groups (*P* = 0.014 and *P* < 0.001, respectively). Children with SMA had the highest carriage of heterozygous and homozygous α^3.7^-thalassemia variants, the lowest inheritance of HbAS, and the highest proportion of HbSS. The distribution of glucose-6-phosphate dehydrogenase (G6PD) deficiency was comparable across the groups (*P* = 0.387).

### Characteristics of the *NCR3* variants in the study population

**Fig. 1:**
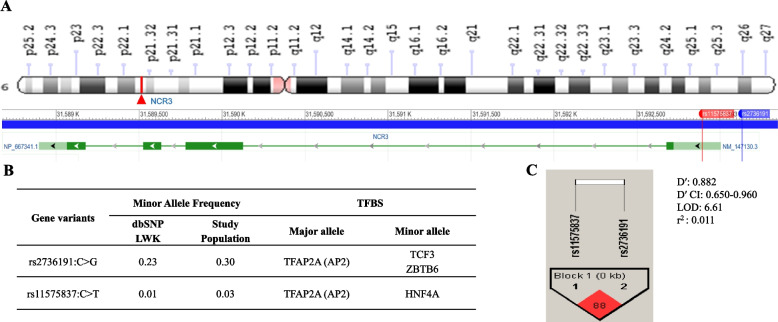
Chromosome location of *NCR3* and linkage disequilibrium of SNPs under investigation. **A** Human natural cytotoxicity-triggering receptor 3 gene (*NCR3*) is located on chromosome 6p21.33 (GRCh38.p12). The SNPs under investigation were rs2736191:C > G and rs11575837:C > T, respectively. rs2736191 is located in the proximal promoter region, while rs11575837 is a 5’ UTR variant and located in exon 1. **B ***NCR3* minor allele frequencies (MAF) for the Luhya (LWK) and Luo populations. Transcription factor binding analyses of the *NCR3* variants. Transcription Factor AP-2 alpha, TFAP2A; Transcription Factor 3, TCF3; Zinc finger and BTB domain containing 6, ZBTB6; and Hepatocyte nuclear factor 4, HNF4A. **C** Linkage disequilibrium between the selected *NCR3* SNPs (D’: 0.882, LOD: 6.61, *r*^2^: 0.011)

*NCR3* is located on chromosome 6p21.33 (GRCh38.p12). The two SNPs selected for investigation, shown in Fig. 1A, have been shown to functionally impact gene expression and clinical outcomes in mild malaria (i.e., rs2736191) and primary pSS (i.e., rs11575837), respectively [30, 31]. Genotyping of the two SNPs in the population revealed a MAF for rs2736191 at 0.30 and rs11575837 at 0.03 (Fig. 1B). Transcription factor binding site (TFBS) analysis, using TFBIND [37], showed that the conversion of C to G in rs2736191 caused ablation of the binding site for AP-2 alpha (i.e., TFAP2A or AP2) and creation of binding sites for transcription factor 3 (i.e., TCF3 or E47) and zinc finger and BTB domain containing 6 (i.e., ZBTB6 or ZID, Fig. 1B). TFBS analysis of rs11575837 revealed that conversion of C to T results in loss of the TFAP2A binding site and the gain of a hepatocyte nuclear factor 4 (HNF4A) binding site (Fig. 1B). LD analysis between the selected SNPs yielded D′ as 0.882, LOD as 6.61, and *r*^2^ as 0.011 (Fig. 1C).

The distribution of genotypes and haplotypes for rs2736191 and rs11575837 is shown in Table 2. The observed frequencies of the rs2736191 genotypes in the overall population were 0.503 for CC, 0.386 for CG, and 0.111 for GG, displaying significant departure from HWE (χ^2^ = 11.58, *P* < 0.001). The observed frequencies of the rs11575837 genotypes in the overall population were 0.941 for CC, 0.054 for CT and 0.005 for TT, displaying significant departure from HWE as well (χ^2^ = 28.57, *P* < 0.001). The distribution of the genotypes and the four haplotypes were comparable across the three clinical groups.

**Table 2:** Distribution of rs2736191:C > G and rs11575837:C > T genotypes and haplotypes

**Genotype/haplotype**	**Total**	**Aparasitemic**	**Non-SMA (Hb ≥ 5.0 g/dL)**	**SMA (Hb < 5.0 g/dL)**	***P-*value**
**No. of participants**	**1,515**	**289**	**962**	**264**	
**rs2736191**					
CC, n (%)	762 (50.3)	153 (52.9)	490 (50.9)	119 (45.1)	0.280
CG, n (%)	585 (38.6)	101 (35.0)	372 (38.7)	112 (42.4)	
GG, n (%)	168 (11.1)	35 (12.1)	100 (10.4)	33 (12.5)	
HWE (χ^2^, *P*)	11.58, < **0.001**				
**rs11575837**					
CC, n (%)	1,426 (94.1)	272 (94.1)	908 (94.4)	246 (93.2)	0.169
CT, n (%)	81 (5.4)	17 (5.9)	46 (4.8)	18 (6.8)	
TT, n (%)	8 (0.5)	0 (0.0)	8 (0.8)	0 (0.0)	
HWE (χ^2^, *P*)	28.57, < 0.001*				
**rs2736191/rs11575837**					
Non-CC, n (%)	196 (12.9)	38 (13.1)	121 (12.6)	37 (14.0)	0.821
CC, n (%)	1,319 (87.1)	251 (86.9)	841 (87.4)	227 (86.0)	
Non-CT, n (%)	1,426 (94.1)	272 (94.1)	908 (94.4)	246 (93.2)	0.762
CT, n (%)	89 (5.9)	17 (5.9)	54 (5.6)	18 (6.8)	
Non-GC, n (%)	764 (50.4)	153 (52.9)	492 (51.1)	119 (45.1)	0.139
GC, n (%)	751 (49.6)	136 (47.1)	470 (48.9)	145 (54.9)	
Non-GT, n (%)	1,513 (99.9)	289 (100.0)	960 (99.8)	264 (100.0)	0.562
GT, n (%)	2 (0.1)	0 (0.0)	2 (0.2)	0 (0.0)	

Data are presented as proportions [n, (%)] unless otherwise stated for *NCR3* variants. Children were categorized into aparasitemic (*n* = 289), non-SMA (Hb ≥ 5.0 g/dL, *n* = 962) and SMA (Hb < 5.0 g/dL, *n* = 264). Non-SMA and SMA patients are collectively categorized as parasitemic (*n* = 1,226). Statistical significance was determined by Chi-square analysis across groups. *P*-values ≤ 0.050 were considered significant and are indicated in bold

*HWE* Hardy-Weinberg Equilibrium, *χ*^*2*^ Hardy-Weinberg Equilibrium Chi-square

^*^Represents a significant *P*-value after the exact test for HWE

### Longitudinal risk of malaria and SMA episodes for the NCR3 genotypes and haplotypes

The impact of the genotypes and haplotypes on malaria and SMA episodes across the 36-mos. follow-up period was determined by fitting a Poisson regression model selecting for covariates [i.e., age, sex, co-infections (HIV-1 and bacteremia), G6PD deficiency, sickle-cell trait, and alpha-thalassemia] known to influence susceptibility to malaria and SMA. The results of the regression modeling and occurrence of events is shown in Table 3. There were 7,228 malaria episodes, for which 406 SMA events occurred during the cohort observational period. Significant effects on susceptibility to malaria were observed for rs11575837 in which carriage of homozygous recessive genotype (TT) increased the risk of malaria (IRR = 1.540, 95% CI = 1.114–2.129, *P* = 0.009, significant after Holm-Bonferroni correction). Neither rs2736191 nor any of the haplotypes derived from the two SNPs significantly impacted the longitudinal susceptibility to malaria.

**Table 3:** Susceptibility to malaria and SMA over 36 mos. of follow-up

Genotype/haplotype		Malaria episodes				SMA episodes			
		**n**	**IRR**	**95% CI**	***P***	**n**	**IRR**	**95% CI**	***P-*value**
**rs2736191**	CC	3,575	Ref			184	Ref		
	CG	2,832	1.011	0.957–1.067	0.700	170	1.269	1.009–1.597	**0.041**
	GG	821	1.014	0.936–1.100	0.731	52	1.378	0.991–1.915	0.057
	Additive	7,228	1.010	0.974–1.048	0.590	406	1.198	1.030–1.393	**0.019**
**rs11575837**	CC	6,809	Ref			379	Ref		
	CT	370	0.957	0.853–1.074	0.454	24	1.294	0.828–2.020	0.258
	TT	49	1.540	1.114–2.129	0.009^*^	3	1.911	0.609–6.000	0.267
	Additive	7,228	1.031	0.935–1.136	0.540	406	1.330	0.929–1.904	0.119
**rs2736191/rs11575837**									
**CC**	Non-CC	978	Ref			61	Ref		
	CC	2,971	0.976	0.908–1.048	0.503	181	0.850	0.637–1.135	0.271
	Additive	6,250	0.990	0.955–1.026	0.571	345	0.823	0.711–0.952	0.009*
**CT**	Non-CT	6,809	Ref			379	Ref		
	CT	385	0.995	0.893–1.108	0.920	25	1.271	0.839–1.925	0.257
	Additive	419	1.015	0.918–1.123	0.769	27	1.295	0.886–1.894	0.182
**GC**	Non-GC	3,590	Ref			185	Ref		
	GC	2,817	1.009	0.960–1.061	0.726	169	1.276	1.030–1.581	**0.026**
	Additive	3,638	1.007	0.971–1.044	0.709	221	1.182	1.018–1.372	**0.029**
**GT**	Non-GT	7,213	Ref			405	Ref		
	GT	15	1.574	0.947–2.619	0.080	1	1.196	0.166–8.627	0.859
	Additive	15	1.574	0.947–2.619	0.080	1	1.196	0.166–8.627	0.859

Data are presented as incidence rate ratio (IRR) with 95% confidence intervals (CI) using log-linear regression with the following covariates in the models: age at enrollment, sex, HIV-1, bacteremia, sickle cell trait, α^3.7^-thalassemia, and G6PD deficiency. The longitudinal relationship between the genetic variants and susceptibility to malaria and SMA (Hb < 5.0 g/dL) was determined throughout the 36 mos. follow-up period. *P*-values ≤ 0.050 were considered significant and are indicated in bold

^*^P-value remained significant after Holm-Bonferroni correction for multiple comparisons

A similar Poisson regression selecting for the same covariates revealed an increased risk of SMA over 36-mos. in carriers of rs2736191 genotypes CG (IRR = 1.269, 95% CI = 1.009–1.597, *P* = 0.041), and in the additive model (IRR = 1.198, 95% CI = 1.030–1.393, *P* = 0.019). There was a trend of increased risk of SMA in carriers of genotype GG without significance (IRR = 1.378, 95% CI = 0.991–1.915, *P* = 0.057). However, none of the results remained significant after correction for multiple comparisons. Carriage of CT or TT genotypes for rs11575837 showed a non-significant increased risk of developing SMA [(IRR = 1.294, 95% CI = 0.828–2.020, *P* = 0.258) and (IRR = 1.911, 95% CI = 0.609–6.000, *P* = 0.267), respectively]. Analysis of the impact of the haplotypes on susceptibility to SMA revealed an additive protective effect for individuals who inherited the CC haplotype (IRR = 0.823, 95% CI = 0.711–0.952, *P* = 0.009, significant after Holm-Bonferroni adjustment). Conversely, there was an enhanced risk of SMA in individuals who inherited the GC haplotype (IRR = 1.276, 95% CI = 1.030–1.581, *P* = 0.026), and in the additive model (IRR = 1.182, 95% CI = 1.018–1.372, *P* = 0.029), but neither retained significance after Holm-Bonferroni adjustment. There was no association between an altered risk profile of SMA for either the CT or GT haplotypes.

A Cox proportional hazard modeling was fitted to examine the relationship between the *NCR3* genotypes/haplotypes and all-cause mortality. No significant relationships with all-cause mortality were observed for either rs2736191, rs11575837, or their haplotypes (Additional file 1: Table S1).

## Discussion

Nearly every child under 5 years of age in the holoendemic *P. falciparum* transmission area where the study was conducted experience repeated episodes of malaria, yet only a subset of individuals develop SMA, typically within the first year of life. Young children (< 12 mos.) are especially susceptible to severe malaria as maternal immunity begins to wane and adaptive immunity starts to develop with repeated episodes of malaria [38]. Since childhood immunity to malaria gradually develops across successive episodes [39], the impact of genetic variants of malaria and SMA is best captured in longitudinal studies during the development of naturally-acquired malarial immunity [40]. To the best of our knowledge, we report the first study on the longitudinal risk of clinical malaria and SMA in carriers of rs2736191 and rs11575837, and their haplotypes.

The SNPs targeted for investigation were selected based on previous reports showing that the variants impart functional changes in gene expression and influence mild malaria and pSS [19, 20, 30, 31]. Notably, the substitution of C by G in rs2736191 creates TFBSs for TCF3 or ZBTB6 and ablates a binding site for TFAP2A (AP2). Previous studies show that TFAP2A (AP2) regulates the transcription of IFN-γ Receptor 1 (IFNGR1), and that elevated IFN-γ production is protective against infection with malaria [41–43]. In addition, TCF3 was shown to promote the development of γδ T cells, differentiation of memory CD8 T cells, and increase γδ T cells and CD8 + T cell responses in children with SMA [44–47]. Although not reported in malaria, human *ZBTB6* was one of the most differentially expressed genes in the whole blood of patients with Crohn’s disease [48]. Conversion of C to T in rs11575837 leads to the loss of a TFAP2A (AP2) binding site and the gain of a HNF4A binding site. Although HNF4A was shown to promote erythropoiesis during embryonic development in a murine model, it remains to be determined if children who are carriers of the T-allele have altered binding to HNF4A and subsequent alterations in erythropoiesis [49].

The MAFs of the variants under investigation were higher in the study participants than for the LWK ethnic group included in the 1000 Genome Project [31]. Additionally, the overall distribution of rs2736191 and rs11575837 genotypes in our study population displayed significant departure from HWE, indicative of an influence of evolutionary forces on the human genome in the study population. There is mounting evidence that natural selection influences the frequencies of disease-associated genetic variants in different populations [50]. For example, malaria exerts a strong evolutionary force on risk-associated alleles [7, 40] and may, at least in part, explain findings presented here on the allelic distribution.

LD analysis revealed that the two SNPs are co-inherited (D′ = 0.882). Since D′ values are known to fluctuate upwards for less common alleles (i.e., rs11575837), we also determined the D′ confidence intervals and *r*^2^ [51]. D′ captures the recombination events (inheritance) between an allele of one SNP and that of another, while *r*^2^ is a statistical measure of correlation between the SNPs, therefore, both should be considered when deciphering the degree of association between SNPs [52]. Thus, both SNPs were investigated since the D′ was high and the *r*^2^ value was low (*r*^2^ = 0.01), suggesting that the two SNPs may convey different information.

Although previous studies found an association between carriage of the minor allele for rs2736191 and increased mild malaria [19, 20], there was no relationship between the different genotypes and malaria episodes in our study population. In the former investigation in Congolese children [20] the relationship between the mutant allele and mild malaria was only significant in children greater than 5 years of age, but not in younger children [20]. Moreover, in the parent-sib study conducted in Burkina Faso, the mean age of the sibs was 12.1 ± 6.2 years [19]. Collectively, these studies suggest an age-specific relationship between carriage of the minor allele and susceptibility to malaria that may not have been detected in our study since the population was considerably younger (primarily < 5 years). Although this hypothesis remains to be tested, there was a significant relationship between carriage of the G-allele and increased susceptibility to SMA in carriers of GG and in the additive model. However, this relationship was not significant after testing for multiple comparisons.

Although the previous family-based association study in Burkina Faso did not include rs11575837 in the analyses due to a low MAF (< 0.01) in the study population, we investigated this SNP based on its association with functional properties and susceptibility to pSS [30, 31]. The MAF in the current study for rs11575837 was 0.03 with TT carriers having significantly increased susceptibility to acute malaria across the follow-up period. The minor allele T has been associated with lowered *NCR3* expression [30]. Since NCR3 can activate NK cells to clear malaria parasites by directly recognizing pRBCs [23, 24, 27], decreased NCR3 expression in TT carriers could reduce antiparasitic effects and result in increased susceptibility to malaria. Thus, even though there was a low MAF for rs11575837 in the study cohort, carriage of both T-alleles did impact on susceptibility to malaria, consistent with results found for other diseases in European populations in which the MAF was also low (0.02) [30, 31].

While there was also an increased risk for SMA (RR = 1.9) in children who inherited TT for rs11575837, the results were not significant, likely due to the low number of events for rare variant. Although requiring further evidence, it is possible that the rarity of the TT genotype maintained in the study population could be explained by increased susceptibility to malaria, SMA, and subsequent mortality. There was a progressive increase in the risk of mortality associated with carriage of T-alleles but the low carriage rate in the context of a low mortality rate cannot confirm this hypothesis. In the study region, childhood mortality from malaria has historically been very high [3]. As such, failure to reach reproductive age has had a strong impact on selection.

Investigations on the impact of coinheritance of the two SNPs did not elucidate any significant findings for altered susceptibility to longitudinal malaria episodes. However, the additive model revealed that a progressive carriage rate of both wild type alleles (CC) significantly reduced the risk of SMA. This finding is consistent with selective effects for the variants in the population, particularly for the rarer rs11575837 mutant allele. Additional modeling efforts revealed that inheritance of the CG genotype increased the risk of SMA in carriers versus non-carriers and in the additive model, but the results were not significant after correction for multiple comparisons. Nonetheless, in the context of the significant risk found for carriage of G-alleles for rs2736191 in the genotypic model, it appears that carriage of the G-allele in the GC haplotype certainly influences the longitudinal development of SMA.

Secondary analyses were performed to determine if the SNPs (or their haplotypes) predict all-cause mortality. Inheritance of either of the two SNPs individually, or in combination, were not significantly associated with all-cause mortality. However, based on the reduced number of carriers for several of the variants in the context of an overall low mortality rate in the cohort, the influence of the selected variants on childhood mortality could not be determined with a high level of statistical confidence. Additionally, there are limitations of the current study: the covariates in the statistical model did not include other potential anemia-promoting factors such as nutrition status, and the observed deviation from the HWE may introduce a bias in a genetic association study [53].

## Conclusions

We provide the first evidence that carriage of the TT genotype for rs11575837 is linked to increased longitudinal malaria episodes. Additional findings include the protective effect of CC haplotype carriage against SMA in the additive model, and conversely, the additive effect of GC haplotype carriage on enhanced susceptibility to SMA. Collectively, these novel findings support further studies to define the molecular mechanisms by which NK cells and their pathways influence the pathogenesis of SMA. Such findings could facilitate the development of improved strategies for the control and clinical management of severe malaria.

## Methods

### Study site and study participants

The prospective observational study over 36 mos. was conducted in children presenting at Siaya County Referral Hospital (SCRH) to determine the relationship between NCR3 variants and susceptibility to malaria and SMA. SCRH is in western Kenya, a region of holoendemic *P. falciparum* malaria transmission [54, 55]. Severe malaria in western Kenya primarily manifests as SMA [56, 57]. Study participants (*n* = 1,515, aged 1.9–40 mos.), who either reported for their first documented hospital visit for febrile episodes or routine childhood vaccinations, were recruited at SCRH. Written informed consent in the language of choice (i.e., English, Swahili or Dholuo) was obtained from the parent or legal guardian of all children participating in the study. Questionnaires were used to collect demographic and clinical information. Based on *P. falciparum* parasite density and Hb levels in peripheral blood, study participants were grouped into three categories upon enrollment: aparasitemic (*n* = 289), non-SMA (Hb ≥ 5.0 g/dL, *n* = 962), and SMA (Hb < 5.0 g/dL, *n* = 264). Exclusion criteria included: children with cerebral malaria (a rare occurrence in this holoendemic area); clinical evidence of acute respiratory infection; and prior hospitalization. Patients were treated according to the Ministry of Health-Kenya guidelines.

### Longitudinal follow-ups

Following enrollment, parents/guardians were requested to bring their child to hospital every three mos. throughout the 36-mo. longitudinal follow-up period and during any acute febrile episodes. A complete physical exam and clinical laboratory tests were performed at each quarterly and acute visit. All-cause mortality data was collected throughout the follow-up period. The geographic information system coordinates of each child’s address were recorded upon enrollment. For the participants who did not report for a scheduled follow-up visit, our study field team went to the residence to determine the health status of the child, which included a verbal autopsy in cases of mortality.

### Laboratory investigations

Heel or finger-prick blood samples (< 100 μL) were obtained and used to determine key variables such as parasitemia and Hb concentrations according to previous published methods [54]. Complete blood counts (CBC) were assessed using the Beckman Coulter ACT diff2™ (Beckman-Coulter Corporation, Miami, FL, USA). To account for the common causes of severe anemia in the region, anemia-promoting conditions including HIV-1, bacteremia, HbAS status, α^3.7^-thalassemia, and G6PD deficiency were determined. Pre- and post-HIV test counseling was provided to the parents/guardians of all participants. HIV-1 exposure was determined serologically (i.e., Unigold™ and Determine™) and HIV-1 infection was determined by pro-viral DNA PCR testing according to our previous methods [32]. Bacteremia was determined according to our published methods [33]. The presence of the HbAS trait was determined by cellulose acetate electrophoresis as per manufacturer’s instructions (Helena Bio-Sciences, Oxford, United Kingdom), α^3.7^-thalassemia deletion was determined using a PCR-based method [58], and G6PD deficiency was determined by a fluorescent spot test using the manufacturer’s methods (Trinity Biotech Plc., Bray, Ireland).

### Genotyping of rs2736191 and rs11575837 variants

Genomic DNA was extracted from buccal swabs using the MasterAmp™ Buccal swab DNA Extraction kit (Epicentre Biotechnologies, Madison, WI, USA) and amplified using GenomiPhi® system (GE Healthcare, 174 NJ, USA) before genotyping. rs2736191 and rs11575837 were genotyped using our established PCR conditions [15] and the TaqMan^®^ 5’ allelic discrimination Assay-By-Design high-throughput method according to the manufacturer’s instructions [Assay ID: C_16286876_10 for rs2736191 and C_27834902_10 for rs11575837; Thermofisher Scientific, Carlsbad, CA, USA). StepOne™ Software (Version 2.3) was used for allelic discrimination (Thermofisher Scientific, Carlsbad, CA, USA).

### Data analysis

Demographic, clinical and laboratory characteristics of participants at enrolment were analyzed using SPSS^®^ v23.0 (IBM SPSS Inc., Chicago, IL, USA). Data across the study groups was compared using Pearson’s Chi-square (χ^2^) test and the Kruskal-Wallis test. Differences in parasitological variables between SMA and non-SMA were computed using Mann–Whitney U test. Haplotypes composed of rs2736191 and rs11575837 were constructed using HPlus software program v2.5 (Fred Hutchinson Cancer Research Center, Seattle, WA, USA). Proportions of alleles, genotypes, and haplotypes of the *NCR3* variants were compared across the study groups using χ^2^ tests of homogeneity. Hardy–Weinberg Equilibrium (HWE) was evaluated using a χ^2^ goodness-of-fit test for the genotype frequencies of both SNPs. Since there was a low minor allele frequency (MAF) for rs11575837, the exact test for HWE was used [59]. Linkage disequilibrium (LD) was calculated using Multiallelic Interallelic Disequilibrium Analysis (MIDAS) software version 1.0 [60].

The association between *NCR3* genotypes/haplotypes and longitudinal clinical outcomes were analyzed in R (version 3.6.1) by fitting a Poisson rate regression using a forward-backward model selected based on the Akaike Information Criterion (AIC). The following covariates were subject to model selection: age, sex, co-infections (HIV-1 and bacteremia), G6PD deficiency, sickle-cell trait, and alpha-thalassemia, since these covariates can influence malarial anemia [32, 33, 61, 62]. The Poisson regression, accounted for the varying length of the follow-up observational period by treating the logarithm of that length as an offset to the logarithm of the expected number of events (Poisson rate regression). In the “additive model” the Poisson rate regression was carried out by coding the *NCR3* genotypes/haplotypes metrically (indicating 0, 1, or 2 copies of the mutant allele or haplotype, respectively) rather than categorically. Survival analyses were performed by a non-parametric Cox proportional hazard model to determine the relative risk of all-cause mortality associated with *NCR3* genotypes/haplotypes over a 36-mos. follow-up period. For each model, Holm-Bonferroni correction was applied to control for familywise error rates in multiple comparisons. The statistical significance for all analyses was set at *P* ≤ 0.05.

### Supplementary Information

**Additional file 1: Table S1.** Survival analysis for all-cause mortality.

## Data Availability

The data that support the results of this research are available at clinVAR (https://www.ncbi.nlm.nih.gov/clinvar/). The public data can be found at: accession numbers SCV002762723 for rs11575837, and SCV002762724 for rs2736191.

## Abbreviations

SMA: Severe malarial anemia
NCR3: Natural cytotoxicity-triggering receptor 3;
HbS: Sickle hemoglobin
HBB: Hemoglobin beta
MHC: Major histocompatibility complex
NK cell: Natural killer cell
pRBCs: Parasitized red blood cells
DBL1-α: Duffy Binding Like
*Pf*EMP-1: *P. falciparum* Erythrocyte membrane protein-1
pSS: Primary Sjögren’s syndrome
SCRH: Siaya county referral hospital
CBC: Complete blood counts
HbAS: Sickle-cell trait
G6PD: Glucose-6-phosphate dehydrogenase deficiency
HWE: Hardy–Weinberg equilibrium
MAF: Minor allele frequency
LD: Linkage disequilibrium
MIDAS: Multiallelic interallelic disequilibrium analysis
AIC: Akaike information criterion
TFBS: Transcription factor binding site
ZBTB6: Zinc finger and BTB domain containing 6
HNF4A: Hepatocyte nuclear factor 4
