# Supplementary material for: Human NCR3 gene variants rs2736191 and rs11575837 alter longitudinal risk for development of pediatric malaria episodes and severe malarial anemia
Source: BMC Genomics. 2023 Sep 13;24:542. doi: 10.1186/s12864-023-09565-1 (PMC10498606; doi:10.1186/s12864-023-09565-1)
Supplement: Supplementary file 1 — Additional file 1: Table S1. Survival analysis for all-cause mortality. [file 12864_2023_9565_MOESM1_ESM.docx]

**Table S1.** Survival analysis for all-cause mortality.

| **Genotype/Haplotype** | | **Mortality** | | | |
| --- | --- | --- | --- | --- | --- |
|  |  | **n** | **HR** | **95% CI** | ***P-*value** |
| **rs2736191** | CC | 46 | Ref |  |  |
|  | CG | 35 | 1.480 | 0.865-2.520 | 0.153 |
|  | GG | 7 | 0.934 | 0.354-2.460 | 0.890 |
|  | Additive | 88 | 1.140 | 0.783-1.650 | 0.502 |
| **rs11575837** | CC | 83 | Ref |  |  |
|  | CT | 3 | 1.130 | 0.348-3.650 | 0.842 |
|  | TT | 2 | 2.860 | 0.371-22.10 | 0.313 |
|  | Additive | 88 | 1.430 | 0.630-3.260 | 0.390 |
| **rs2736191/rs11575837** | | | | | |
| **CC** | Non-CC | 9 | Ref |  |  |
|  | CC | 37 | 1.410 | 0.604-3.280 | 0.428 |
|  | Additive | 79 | 0.871 | 0.608-1.250 | 0.452 |
| **CT** | Non-CT | 83 | Ref |  |  |
|  | CT | 4 | 1.310 | 0.472-3.630 | 0.605 |
|  | Additive | 5 | 1.240 | 0.467-3.310 | 0.664 |
| **GC** | Non-GC | 47 | Ref |  |  |
|  | GC | 34 | 1.300 | 0.778-2.170 | 0.318 |
|  | Additive | 41 | 1.090 | 0.753-1.590 | 0.641 |
| **GT** | Non-GT | 88 |  |  |  |
|  | GT | 1 | 5.430 | 0.688-42.80 | 0.108 |
|  | Additive | 1 | 5.430 | 0.688-42.80 | 0.108 |

Data are presented as hazard ratios (HR) with 95% confidence intervals (CI). Analysis was performed using Cox regression modeling with the following covariates: age at enrollment, sex, HIV-1 and bacteremia status, sickle cell trait status, α^3.7^-thalassemia, and G6PD deficiency. Homozygous wild-type genotypes and non-carriers of haplotypes were used as reference groups in the analyses. Statistical significance was set at *P≤*0.050. *Significant after Holm-Bonferroni correction for multiple comparisons.
